# Visual Snow: A Review on Pathophysiology and Treatment

**DOI:** 10.3390/jcm12123868

**Published:** 2023-06-06

**Authors:** Przemysław Rusztyn, Wiktoria Stańska, Anna Torbus, Piotr Maciejewicz

**Affiliations:** 1 Students’ Scientific Club, Department of Ophthalmology, Medical University of Warsaw, 02-005 Warsaw, Poland; 2 Department of Ophthalmology, Medical University of Warsaw, 02-005 Warsaw, Poland

**Keywords:** visual snow, visual snow syndrome, neuro-ophthalmology

## Abstract

Objective: Visual snow (VS) is a rare clinical entity in neuro-ophthalmology. It is described as the presence of flickering dots affecting the whole visual field, often compared by patients to snow or pixelated television static. Importantly, it can be an alarming symptom for many patients, lowering their quality of life. Our purpose is to increase awareness of this disease, because many healthcare professionals have difficulty identifying symptoms as the nature of the condition is subjective. In this review, we aimed to describe the updates in the etiology and treatment of visual snow. We searched for articles in English, presenting original data and published after December 2019. Different studies show inconsistent data. Neuroimaging studies found, among other things, hypermetabolism of the lingual gyrus, increased gray matter in different brain areas, and altered connectivity in visual pathways. However, these findings were not present in all patients. According to the literature, among the most effective drugs is lamotrigine. Unfortunately, it also carries a risk of worsening the symptoms. It is crucial to remember that VS can be worsened or induced by alcohol, recreational drugs, and certain medication. In terms of treatment, nonpharmacological approaches such as color filters and repetitive Transcranial Magnetic Stimulation were also used. Interpretation: Further studies are needed to understand the nature of VS fully. Even though the pathophysiology and effective treatment of the condition remains unknown, expanding the knowledge about visual snow can impact the comfort of patients.

## 1. Introduction

Visual snow (VS) is a rare clinical entity described as the bilateral presence of dynamic, flickering dots affecting the whole visual field, often compared to snow or pixelated television static. It usually manifests as white and black dots but can be colorful, transparent, or flashing as well [1]. Its perception varies from being more noticeable on a plain background and diminished on textured backgrounds. In the past, the condition was perceived as a persistent visual aura [2]. According to the current literature, it is a distinct entity [3]. Interestingly, visual snow patients often do not have any abnormalities in ophthalmic examination.

VS can be a lifelong condition, as some patients claim they have had VS since they remember. However, acute-onset VS is also encountered. Moreover, it may be a primary or secondary condition induced by medication or recreational drugs.

What is significant is that VS can be an alarming symptom for many patients, lowering their quality of life. This review article aims to describe the updates in the etiology and treatment of VS. We also aim to increase awareness of this disease because many healthcare professionals have difficulties diagnosing it, as it is a relatively new term in medicine, and the nature of the condition is subjective and elusive.

### 1.1. Additional Visual Symptoms

VS is often accompanied by additional symptoms: non-visual and visual. Among the latter, we can distinguish those of neurological origins, such as:

Palinopsia—the persistence of images beyond the time of exposure. This is suggested to be the result of abnormal persistence of visual memory;

Photophobia—which causes a painful sensation in response to light stimulus;

Nyctalopia—defined as impaired night vision due to dysfunction in the interaction between rod and cone receptors, linked to dysregulation of visual inputs [4].

Visual symptoms arising from the optic apparatus are called entopic phenomena, and they are enhanced in the course of VS. Within the spectrum of enhanced entopic phenomena are floaters, spontaneous photopsia, blue field entopic phenomenon, and self-light of the eye, described by patients as swirls or luminous clouds in their vision [3].

If visual snow is present for longer than three months and there are at least three additional visual symptoms, it is consistent with the diagnostic criteria of visual snow syndrome (VSS) proposed by Schankin et al. [5].

### 1.2. Non-Visual Symptoms

The literature also reports non-visual symptoms, including migraine, occurring in 30% to 60% of visual snow patients. Its prevalence in this group is twice as high as in the general population. Moreover, VSS patients are more likely to have migraine with visual aura [6].

Tinnitus is another common comorbid symptom. It is typically high-pitched and continuous, resulting from disordered auditory pathways. It was proposed that tinnitus is caused by central spontaneous activity in subcortical auditory pathways, normally suppressed. Focusing on the condition may even increase tinnitus. Among other symptoms, phonophobia, hyperacusis, cutaneous allodynia, and even tremors were also found [7].

VS can be a distressing condition for many patients, severely affecting daily life. Moreover, a poor understanding of the disease’s etiology and the subjective nature of the condition may cause psychological discomfort. Studies showed concentration difficulty, lethargy, depression, anxiety, and balance disorder [8,9]. An interesting matter is whether these psychiatric entities are the cause or a result of VS. The frustration resulting from the difficulty of finding the proper healthcare professional, extensive examinations, and management may aggravate the patient’s well-being and mental state. Moreover, it is crucial to remember that labeling VS as purely psychogenic is stigmatizing, weakens the patient’s trust, and even enhances frustration as the patient may blame themself for having the condition.

### 1.3. Differential Diagnosis

VSS is very often underrecognized and, as a result, misdiagnosed with other conditions. A detailed history is crucial in the diagnostic process, as no tests are yet available to confirm it. Emphasis should be placed on neuro-ophthalmology conditions because although the VSS is primary in the majority of cases, it occasionally can be secondary to other disorders. Possible secondary causes of VSS were presented by E. Barral et al. [10]. When the onset is rapid, the differential diagnosis should include bilateral optic neuropathies, e.g., methanol intoxication, ischemia, Leber optic neuropathy, and folate/vitamin B12 deficiency. Other alarming features of the disorder’s picture that should prompt deepening the diagnosis are new onset, particularly at an older age, intermittent or sudden exacerbations, unilateral or hemifield VS, absence of additional visual symptoms, and a history of ophthalmic and neurological conditions. Asking patients about taking recreational drugs may be sensitive. However, it is vital in distinguishing VS from another condition of the visual snow spectrum, hallucinogen persisting perception disorder (HPDD), which may resemble VSS but has a different initiation mechanism [10]. The patient should tell the practitioner if VS is continuous or episodic and if they can recollect a specific event that preceded the onset. Valuable information is whether they have additional visual or non-visual symptoms and which triggers intensify the condition. Careful care is required for patients with a history of migraine, particularly with aura, both visual and non-visual. In migraineurs, VS can be present episodically as a part of the aura, and so it is imperative not to confuse these two different conditions [10].

## 2. Materials and Methods

This review aims to describe the updates in the etiology and treatment of VSS. We followed up on a review about VSS by Eren and Schankin [11], published in May 2020. We searched for articles in English, presenting original data and published from December 2019 to August 2022. We searched for articles on Pubmed by using the following keywords: “visual snow”, “visual snow syndrome,” “visual static,” and “persistent positive visual phenomena” (Table 1).

## 3. Results

The summary of our results regarding pathophysiology is shown in Table 2 and treatment in Table 3.

## 4. Discussion

### 4.1. Pathophysiology

The pathophysiology of VSS remains unknown. A review we are following up on discussed different possible mechanisms and locations of the regions responsible for VSS. It assumes that since VS covers the whole field of vision, its origin cannot be prior to the lateral geniculate nucleus, where the fusion of visual input from both eyes takes place. Instead, it is rather a processing than a structural problem because of the heterogeneous symptoms comprising VSS [11]. This correlates with the results of a trial that performed a couple of neuro-ophthalmologic tests and showed that in 20 patients with VSS, in most cases, there were no abnormalities at all [8]. It also assessed pupillary light reflex in VSS, and in contrast to severe migraines, no abnormalities were shown, even though these diseases are believed to share some pathophysiological mechanisms [8].

Another approach to explaining VSS pathophysiology is using neuroimaging studies. A case series of three patients who underwent SPECT showed that VSS is a heterogeneous disease [25]. Eren and Schankin discussed hypermetabolism of the right lingual gyrus (known for visual postprocessing) in PET and alternations in MRI (r/ADC) in the left occipital cortex [11]. New publications show more possible changes. A case report of VSS after repetitive mild traumatic brain injury showed hypometabolism in the posterior parietal lobes and left posterior cingulate gyrus [29]. A study on the correlation between functional and structural changes in VSS showed hypermetabolism in the right lingual gyrus and hypometabolism in the right superior temporal gyrus and the inferior parietal lobule. Based on this, VBM was performed with regions of interest corresponding with changes in PET. Increased gray-matter volume was found in the lingual gyrus–fusiform gyrus junction. However, no changes were found in the regions of hypometabolism. VBM was then used to perform a whole-brain analysis and found additional grey matter volume increase in the right middle temporal gyrus, the right parahippocampal gyrus, the left superior temporal gyrus, and the right anterior cingulate cortex and a decrease in the left superior temporal gyrus. Unfortunately, patients and controls differed significantly in comorbid migraine, and some of the presented changes occur in migraine. However, the changes can explain VSS’s coexistence with palinacousis, tinnitus, and irritability [24]. Two other studies also used VBM. The first of them showed, similarly to the one above, increased grey-matter volume in the right lingual gurus with a positive correlation between changes in both lingual gyri and the duration of the symptoms with no correlation to their severity [12]. The other also assessed the cerebellum using a high-resolution atlas template SUIT. It showed changes only in gray matter in entirely different areas. They involved the left primary and secondary visual cortices, the left visual motion area V5, and the left cerebellar crus I/lobule VI areas. The changes in the cerebellum might represent a broader network-like disorder, but when corrected for age, sex, handedness, and duration of disease, were not significant [19]. Another argument for cerebellar participation in VSS pathophysiology is shown in a case report describing a transformation from episodic to chronic VSS after infarction of the right superior cerebellar artery territory [23].

Other functional neuroimaging studies also showed changes in VSS. Whole-brain maps of functional connectivity at rest and during VS-like stimulus were acquired with fMRI. Statistical analysis showed that VSS patients have altered connectivity in the pre-cortical and cortical visual pathways, the visual motion network, the attentional networks, and the salience network. The authors suggest that this is due to a disruption in the filtering and integrating of incoming visual stimuli versus internally generated visual information modulation [22]. Another study used whole-brain voxel-wise analysis of BOLD activity and MR spectroscopy of the right lingual gyrus (it was chosen as a key associative visual processing region). There was a significant reduction in BOLD responses in VSS patients compared to healthy controls in both anterior insulas when they saw a film mimicking VS compared to baseline. Additionally, there was a significant increase in lactate concentration in MRS in VSS patients, and a negative correlation between these two parameters was confirmed. This may prove that VSS results from salience network and visual association cortex dysfunction [20]. One more study used fMRI; however, it was performed only at rest. Alternations in the extrastriate visual, frontal, and temporal cortex were shown in regions believed to be related to visual processing, memory, spatial attention, and cognitive control. Despite the absence of visual stimuli, visual processing dysfunction was shown, which correlates with the worsening of symptoms in darkness in some VSS patients [12].

Regarding the changes in DTI-based parameters, a case report shows abnormalities in the dorsal, ventral, and integrative visual streams, acoustic and optic radiations, and thalamic radiations distal to the thalamus. This supports the hypothesis that VSS is unrelated to an eye disease or pure cortical activation and excludes the thalamus from being directly responsible for VSS. However, the changes were diffused [17]. More prominent research regarding DTI-based parameters was performed on a bigger group; however, the DTI only included regions inferior to the body of the corpus callosum. It showed different changes from those above. The distribution of the changes was similar in both papers, but only some of the regions were checked in both. Findings were additionally corrected for interictal migraine occurrence, and fewer differences were significant. When additionally corrected for tinnitus, lateralization to the right side was shown, and consistent changes were seen in the inferior frontal-occipital fascicle, sagittal stratum, and right superior longitudinal fascicle [18]. A high-resolution structural and quantitative MRI showed no differences in morphometry that survived multiple comparison corrections. However, quantitative T1 values were significantly lower for the entire cortical GM, thalamus, pallidum, putamen, and WM for VSS patients, which may represent changes in microstructure in these regions. The authors claim they may affect higher neurite density, myelination, or increased iron levels. When the changes were studied separately for different cortical regions, cortical layers, and individual thalamic nuclei, lower T1 values were seen in most cortical regions in a caudal–rostral pattern, with stronger affection of caudal areas and in most thalamic nuclei. No changes were seen in the inferior temporal cortex, frontal pole, orbitofrontal cortex, insula, and precentral gyrus. No significant changes were found when comparing VSS patients with and without migraine [28].

Regional cerebral blood flow (rCBF) was assessed using arterial spin labeling in VSS patients. It showed that rCBF was higher than controls in many areas, irrelevant to being at rest or looking at a visual-snow-like stimulus. Some of them were bilateral cuneus, precuneus, supplementary motor cortex, premotor cortex, posterior cingulate cortex, left primary auditory cortex, left fusiform gyrus, and left cerebellum, which are mainly involved in complex sensory processing. Moreover, during visual stimulation, rCBF was increased in VSS in the right insula, suggesting a difference in interoceptive processing with a constant perception of altered visual input [21].

Eren and Schankin discussed thalamocortical dysrhythmia as a possible cause of VSS [11]. Another study showed two spectral regions which relieved (orange-yellow and turquoise blue) and one (blue-violet) which exacerbated VSS symptoms, while no controls had any region causing “visual discomfort”. It is conjected that the reported region selectively increases S-cone excitation levels, which results in koniocellular (KC) pathway modulation. KC cell activity increases activity in parvocellular and magnocellular pathways, resulting in conscious awareness of sub-threshold visual stimuli, which causes VSS symptoms [15]. Additionally, magnetoencephalography was used to check the thalamocortical dysrhythmia hypothesis. However, the study’s methods were insufficient to measure subcortical activity and focused on dysrhythmias measured from the early visual cortex. The only significant changes were greater gamma power and reduced alpha-gamma phase–amplitude coupling in VSS patients. They may be evidence of hyperexcitability, potential excitation–inhibition imbalance, and disorganization of noise-canceling mechanisms of the visual cortex, which may support this hypothesis [16].

A different attempt at explaining VSS involves electrophysical examination. Although they show incoherent data, Eren and Schankin discuss visually evoked potential examination findings. One prospective study shows possible dysfunction in the visual association cortex with no changes in the primary visual cortex (normal P100 times and VEP habituation). However, in one case report, potentiation of VEP was shown, and in two prospective studies, loss of habituation was observed, which suggests a dysfunction of the primary visual cortex. They also mention that some patients with VSS had lowered phosphene threshold in the left occipital lobe tested with transcranial magnetic stimulation, and some had reduced gamma-band power in EEG [11]. Another electrophysical test checked if reduced magnetic suppression of perceptual accuracy (MSPA) occurred in VSS as observed in chronic migraine and episodic migraine with aura. In contrast to these diseases, inhibition of the primary occipital cortex, as assessed with MSPA, may not be affected in VSS. This means that even though VSS is believed to be caused by cortical hyperexcitability, it probably does not involve the primary visual cortex [13].

However, behavioral data acquired with visual tasks, which claim different central areas for encoding and processing, showed a possible dysfunction of the primary visual cortex. In contrast, the association visual cortex remained unaffected [11]. Another study on VSS’s behavioral aspect showed that patients with VSS had longer PS task latency and made more errors in the AS task but showed no significant changes in the interleaved AS-PS task. This suggests that the underlying problem is speeded PS response due to changes to cortical processing in VSS [26]. The same group of authors suggested that these changes are due to faster exogenous attention shifting and showed that endogenous attention shifting is also affected using two saccade tasks. VSS patients generated significantly more errors in both. Valid trial latencies were significantly shorter, and cue effect was significantly larger, in the endogenously cued saccade task [27]. Another study conducted by the same authors was an ocular motor version of Posner’s “Inhibition of Return” (IOR) paradigm. It showed that compared to healthy controls, IOR was observed in longer stimulus onset asynchronies. This suggests an imbalance between facilitatory and inhibitory saccade activity in VSS. However, no group effect was shown regarding the error rate. The authors suggest that the differences likely result from altered activation within early visual processing regions and/or disruption to thalamocortical networks, particularly attentional networks [14]. The last three cited articles claim to present a saccadic behavioral profile which may be an objective measure of dysfunction to monitor the efficiency of future treatments and help in future research [14,26,27].

The last attempt at explaining the pathophysiology of VSS by Eren and Schankin was analyzing the whole clinical picture and assuming that it may be due to stochastic resonance, which could explain the occurrence of tinnitus and photophobia [11]. No new studies concerning this aspect were found.

Unfortunately, it is impossible to conclude on the exact pathophysiology of VSS, as different studies show inconsistent data. However, many different regions may be affected, which confirms that it is a very complex condition. Inconsistent changes in many different neuroimaging areas suggest that finding a structural abnormality responsible for VSS is probably unachievable. It seems that it is a network-like disorder in which sensory processing and attention are affected, resulting in awareness of sub-threshold stimuli that are usually omitted. Further research considering this aspect is needed. Analysis of the exacerbating and relieving factors and the exact symptomatology in larger populations may be helpful. Additionally, a more accurate analysis of patients with VSS regarding their comorbidities, psychological profile, personality traits, and exposure to stress should be undertaken. Some of the shown data might be used to diagnose and monitor patients as no objective method of subjective assessment of this disease is currently available.

### 4.2. Treatment

There is no effective treatment that can be used to improve VSS. Since this syndrome’s pathophysiology remains unknown, mechanism-based therapy or randomized controlled trials cannot be performed. Our knowledge about possible treatment options comes from case reports, case series, and retrospective cohort studies; prospective studies are very rare. Not all reports mention timespans or even doses of medication, and other treatments are described only by drug categories. These factors make the comparison between studies difficult. Only a minority of patients benefit from taking medication. Anticonvulsants were most frequently tried in the case of reports, with the highest efficacy for lamotrigine [11]. The survey conducted by Puledda et al. did not confirm this trend, where the improvement ratio of lamotrigine was not significantly higher than other medications and treatment caused a worsening in more cases [32]. This study showcases that no drug had a sufficient improvement rate. This work also mentions substances such as recreational drugs and alcohol that should be avoided in patients with VSS because they tend to worsen symptoms. Medications that should be used cautiously because of the high risk of worsening are atypical antidepressants and ADHD drugs. One work describes full recovery from VSS by the shift from Methylphenidate to Atomoxetine in ADHD treatment [35]. It is essential to highlight that not all patients are willing to try pharmacological treatment for VSS, possibly due to the fear of side effects, mainly worsening visual symptoms. These factors may increase the importance of nonpharmacological VSS treatment. Repetitive Transcranial Magnetic Stimulation is a relatively new method; it remains controversial whether this non-invasive brain stimulation can be applied to the visual cortex, but recent studies include the usage of rTMS as an effective therapy in reducing VS intensities if 10 + 1 Hz was applied [30]. Another effective nonpharmacological approach is color filters [11], especially from the yellow-blue color spectrum or FL-41 glasses effective for photophobia [31].

## 5. Conclusions

We are still far from fully understanding the pathophysiology of VSS. Imaging studies describe many different abnormalities, but none occur in all patients which explain all the symptoms. The behavioral and electrophysiological examination also did not deliver a clear explanation of the origin of this disease. The multitude of detected changes proves that it is probably a multifactorial disease. Lamotrigine is considered the drug with the best-proven effect, but it is essential to remember that it can cause the worsening of symptoms. Nonpharmacological treatments that might benefit patients include rTMS sessions and tinted glasses for everyday use. Without more research, the best action we can provide is to support patient adaptation mechanisms.

We believe that by expanding the knowledge about VSS, we can have a real impact on the comfort of patients. That is why ophthalmologists and neurologists should be able to diagnose VSS, as a quick and proper diagnosis lets patients avoid stress and shortens the diagnostic path.

## Figures and Tables

**Table 1:** Criteria used for article searching.

Inclusion Criteria	Exclusion Criteria
Articles presenting original data Articles published after December 2019 All patients must fulfill VSS criteria or there is a cohort of patients that fulfill them that is considered separately Articles in English	Articles about Hallucinogenic Persisting Perception Disorder

**Table 2:** Studies on the pathophysiology of VSS (alphabetical order).

Reference Number	Patients with VSS (Mean Age)	Controls (Mean Age)	Methods	Results
[12]	19 (33.3)	16 (31.6)	Original research: fMRI with an assessment of resting-state functional connectivity (rsFC) Voxel-based morphometry (VBM)	Hyperconnectivity between extrastriate visual and inferior temporal brain regions and between prefrontal and parietal brain regions. Increased grey matter volume in the right lingual gyrus in VSS.
[13]	17 (30.0)	17 (28.3)	Original research: Reduced magnetic suppression of perceptual accuracy (MSPA)	Occipital cortex inhibition assessed with MSPA is not affected in VSS.
[14]	40 (26.7)	20 (25.6)	Original research: An ocular motor version of Posner’s “Inhibition of Return” (IOR) paradigm	Delayed onset of IOR in VSS.
[15]	22 (31.8)	12 (38.4)	Original research: Intuitive Colorimetry testing.	Symptoms are exacerbated by color modulation that selectively increases levels of S-cone excitation.
[16]	18 (29)	16 (31)	Original research: Magnetoencephalography	Significantly higher gamma (40–70 Hz) power in the primary visual cortex and reduced phase-amplitude coupling—hyperactivation and disorganization of cortical activity during early visual processing.
[17]	1 (28)	10 (26)	Case report: Diffusion tensor imaging (DTI)-based parameters	Abnormalities in the dorsal, ventral, and integrative visual streams, and acoustic, optic, and thalamic radiations.
[18]	14 (32)	20 (28.2)	Original research: DTI-based parameters	Abnormalities in prefrontal, temporal, and occipital white matter, superior and middle longitudinal fascicle, and sagittal stratum. When additionally corrected for tinnitus or migraine, less pronounced.
[19]	24 (28)	24 (28)	Original research: MRI with voxel-based morphometry analysis of cerebral and cerebellar anatomy.	Increased gray matter volume in the left primary and secondary visual cortices, left visual motion area V5, and the left cerebellar crus I/lobule VI area.
[20]	24 (28)	24 (28)	Original research: Blood oxygenation level-dependent (BOLD) fMRI during visual-snow-like stimulus Proton Magnetic Resonance Spectroscopy (H-MRS)	Reduced BOLD responses to the visual stimulus in both anterior insulas in VSS. Significant increase of lactate concentrations in the right lingual gyrus in VSS.
[21]	24 (28)	24 (no data)	Original research: MRI with whole-brain maps of regional cerebral blood flow (rCBF) at rest and during visual snow stimulus	Higher rCBF in bilateral cuneus, precuneus, supplementary motor cortex, premotor cortex, posterior cingulate cortex, left primary auditory cortex, fusiform gyrus, and cerebellum at rest and during visual stimulation. Increased rCBF in the right insula during visual stimulation.
[22]	24 (28)	24 (28)	Original research: fMRI: whole-brain maps of functional connectivity at rest and during visual-snow-like stimulus	Hyper and hypoconnectivity within visual, attentional, and salience networks in VSS.
[23]	1 (44)	NA	Case report: MRI	Infarction of superior cerebellar artery caused a transformation from episodic to chronic VSS.
[24]	20[PET] (31) 17 [VBM] (no data)	20[PET] (30) 17 [VBM] (no data)	Original research: FDG-PET VBM	Corresponding structural and functional changes in visual association cortex. Other broad alterations suggest that VSS extends beyond the visual system.
[25]	3 (26.7)	NA	Case series: SPECT	Different outcomes in all cases: (1) Right occipital and temporal hypoperfusion- ventral visual stream, (2) mild bilateral frontal hypoperfusion, (3) no overt abnormalities
[26]	64 (31.56)	23 (28.74)	Original research: Prosaccade (PS) task Antisaccade (AS) task Interleaved AS-PS task	Visual processing changes in VSS: shortened PS latencies and increased rate of AS errors with no significant changes in AS-PS task.
[27]	67 (30.63)	37 (27,56)	Original research: Endogenously cued saccade task, Saccadic Simon task	VSS patients generated significantly more errors in both tests. Valid trial latencies and cue effect were substantially larger in the endogenously cued saccade task, with no more significant changes in the Saccadic Simon task.
[28]	40 (33.2)	43(29.2)	Original research: High-resolution structural and quantitative MRI	No significant changes in morphometry in VSS patients. Widespread changes in the microstructure of the grey matter—lower quantitative T1 values for entire cortical GM, thalamus, pallidum, putamen and white matter. No significant differences between VSS patients with and without migraine.
[29]	1(40)	NA	Case report: MRI Magnetic resonance angiography Electroencephalography Cerebrospinal fluid analysis PET	Hypometabolism in the posterior parietal lobes and left posterior cingulate gyrus.
[8]	20 (25.4)	NA	Retrospective review: Best-corrected visual acuity (BCVA), automated refraction slit-lamp biomicroscopy, dilated fundus examinations, visual field testing, pupillary light reflex, contrast sensitivity, full-field and multifocal electroretinography, optical coherence tomography with an assessment of the thickness of the retinal nerve fiber layer (RNFL)	Neuro-ophthalmologic tests are mostly normal in patients with VSS.

**Table 3:** Articles that studied the effect of medications in visual snow treatment (in chronological order of publication).

Reference Number	Patients with VSS (Mean Age)	Treatment	Effect on VS
[8]	*n* = 20 [5 treated] (25,4)	Lamotrigine 25 mg/d Topiramate 25 mg/d Acetazolamide 750 mg/d Propranolol 20 mg/d (*n* = 2)	All ineffective
[30]	*n* = 9 (32,6)	Repetitive Transcranial Magnetic Stimulation	10 + 1 Hz reduced sum of VS intensities week after therapy, 10 Hz no significant changes
[31]	*n* = 1 (41)	Phenylephrine, FL-41 glasses	Phenylephrine gives partial response for nyctalopia; FL-41 glasses effective for photophobia
[32]	*n* = 400 (31)	Antidepressants: SSRIs, Tricyclics, Atypical; Vitamins/nutraceuticals; Antiepileptics: Topiramate, Lamotrigine, Gabapentin, Valproic acid, Pregabalin; Antibiotics/antifungals; Benzodiazepines/hypnotics, NSAIDs, Paracetamol/acetaminophen, Opioids, Antihypertensive drugs, Steroids, Triptans, ADHD medication, amphetamine-type, atomoxetine, methylphenidate, Antihistamines decongestants, Antipsychotics, Nausea/dizziness medication	Benzodiazepines/hypnotic, Triptans, Lamotrigine, Tricyclic antidepressants, Gabapentin, Beta-blockers, Topiramate, and Vitamins/nutraceuticals had the best improvement ratio.
[33]	*n* = 10 (no data)	Repetitive Transcranial Magnetic Stimulation	Study discontinued.
[34]	*n* = 1 (15)	Topiramate 25 mg, Amitriptyline 30 mg, Gabapentin 900 mg, Onabotulinium toxin A	All ineffective. Topiramate caused a worsening. Onabotulinium toxin A treatment is ongoing.
[29]	*n* = 1 (40)	Topiramate, Lamotrigine, Sumatriptan, hyperbaric oxygen chamber therapy, tinted glasses	Pharmacological treatment was ineffective. Improving VS with blue medical filters.
[35]	*n* = 1 (10)	Methylphenidate for ADHD was a trigger for VS, then changed to Atomoxetine.	Dose reduction of Methylphenidate causes VS improvement but worsens ADHD symptoms. The shift for Atomoxetine caused improvement in ADHD with no visual phenomena.

## Data Availability

No new data were created or analyzed in this study. Data sharing is not applicable to this article.
